# Ocean acidification influences host DNA methylation and phenotypic plasticity in environmentally susceptible corals

**DOI:** 10.1111/eva.12408

**Published:** 2016-08-02

**Authors:** Hollie M. Putnam, Jennifer M. Davidson, Ruth D. Gates

**Affiliations:** ^1^ Hawaii Institute of Marine BiologyUniversity of HawaiiKaneoheHIUSA

**Keywords:** acclimatization, coral, epigenetics, metabolomics

## Abstract

As climate change challenges organismal fitness by creating a phenotype–environment mismatch, phenotypic plasticity generated by epigenetic mechanisms (e.g., DNA methylation) can provide a temporal buffer for genetic adaptation. Epigenetic mechanisms may be crucial for sessile benthic marine organisms, such as reef‐building corals, where ocean acidification (OA) and warming reflect in strong negative responses. We tested the potential for scleractinian corals to exhibit phenotypic plasticity associated with a change in DNA methylation in response to OA. Clonal coral fragments of the environmentally sensitive *Pocillopora damicornis* and more environmentally robust *Montipora capitata* were exposed to fluctuating ambient pH (7.9–7.65) and low pH (7.6–7.35) conditions in common garden tanks for ~6 weeks. *M. capitata* responded weakly, or acclimated more quickly, to OA, with no difference in calcification, minimal separation of metabolomic profiles, and no change in DNA methylation between treatments. Conversely, *P. damicornis* exhibited diminished calcification at low pH, stronger separation in metabolomic profiles, and responsiveness of DNA methylation to treatment. Our data suggest corals differ in their temporal dynamics and sensitivity for environmentally triggered real‐time epigenetic reprogramming. The generation of potentially heritable plasticity via environmental induction of DNA methylation provides an avenue for assisted evolution applications in corals under rapid climate change.

## Introduction

Phenotypic plasticity is the flexibility for a single genotype to produce a range of responses to biotic and abiotic environmental conditions (Hochachka and Somero 2002). Plasticity in response to the environment provides a dynamic mechanism for generating rapid variability in traits that effect ecological performance and subsequently fitness (Schlichting and Pigliucci 1998). The rapid rate of change in the physical environment driven by anthropogenic climate change (Pachauri et al. 2014) presents a challenge to organismal fitness by creating a mismatch between phenotypes adapted to a more stable or predictable historic environmental and the new conditions. Phenotypic plasticity, therefore, provides a real‐time compensatory response to this rapid environmental change that can act to provide a temporal buffer during which genetic variation can respond under natural selection. Additionally, the interplay of genetic and epigenetic variation results in emergent evolutionary properties that can influence the capacity for organisms to respond to swift environmental change (Ghalambor et al. 2015).

One mechanism of phenotypic plasticity that has the potential to facilitate rapid beneficial acclimatization (Huey et al. 1999; Wilson and Franklin 2002) and is gaining attention across taxa from humans (Egger et al. 2004) to plants (Rapp and Wendel 2005) is epigenetics, or the heritable postsynthesis modification of DNA or DNA‐associated proteins, without a change in the DNA sequence itself (Feil and Fraga 2012). Epigenetic mechanisms provide capacity for the genome to produce multiple outcomes from the same genetic material, *via* changes in gene expression, induced by developmental differentiation (Waddington 1942) and environmental triggering (Bossdorf et al. 2008; Feil and Fraga 2012). Epigenetics *sensu stricto* includes mechanisms such as control on gene expression *via* chromatin modifications (Li 2002; Greer and Shi 2012), DNA methylation (Bird 2002), and small RNAs (Feil and Fraga 2012; Castel and Martienssen 2013). The best studied of these mechanisms to date, however, is epigenetic reprogramming *via* DNA methylation.

DNA methylation is the addition of a methyl group (–CH_3_) to DNA nucleotides, most commonly on cytosine in the sequence CpG in animals. This chemical modification, well studied in vertebrates, results in alteration of access of transcriptional proteins to the promoter regions of DNA (Bird 2002; Suzuki and Bird 2008), thereby influencing transcriptional outcomes. The differential interpretation of the genome through epigenetic mechanisms is therefore accomplished through the silencing, enhancing, and differential splicing of expressed genes, as well as control of spurious intragenic transcription (Bird 2002; Suzuki and Bird 2008; Foret et al. 2012). Epigenetic control of gene expression is heritable by way of maintenance DNA methyltransferase (DNMT1) that propagates hemimethylated DNA during cell replication (Bird 2002; Feil and Fraga 2012). Additionally, DNA methylation is environmentally inducible, occurring in real‐time due to the activity of *de novo* DNA methyltransferase (DNMT3) that initiates novel DNA methylation in response to environmental triggers (Okano et al. 1999; Feil and Fraga 2012). This environmentally responsive mechanism of phenotypic regulation provides one avenue of dynamic phenotypic plasticity that could lead to beneficial acclimatization to changing physical conditions, with the potential for heritability.

DNA methylation patterning is diverse across taxa. Whereas DNA methylation in vertebrates commonly occurs more globally, resulting in silencing of gene expression, in invertebrates, the methylation patterns are more variable, primarily found on gene bodies and exons. Mosaic patterning of DNA methylation in invertebrates is associated with alternative splicing, highlighting different mechanisms of regulation of gene expression between taxa (Feng et al. 2010). Early *in silico* work of predicted genomic methylation in marine invertebrates such as oysters and corals suggests regulation of gene expression by DNA methylation (Roberts and Gavery 2012; Dixon et al. 2014). The depletion of CpG nucleotides in the genome (observed CpG *vs*. expected; CpG_O/E_) provides a signature of consistent historical methylation, as methylated cytosines are hypersensitive to deamination to thymine; predicted methylation based on CpG_O/E_ correlates strongly with empirical measurements (Suzuki et al. 2007; Gavery and Roberts 2013). The examination of predicted methylation in the coral *Acropora millepora* indicates a bimodal distribution of genes, where those with low CpG_O/E_ (strong methylation) are correlated with low expression plasticity and those with high CpG_O/E_ (weak methylation) are correlated with higher expression plasticity (Dixon et al. 2014). Together, the evidence for environmental triggering of *de novo* DNA methylation (Faulk and Dolinoy 2011), the capacity for DNA methylation to regulate gene expression in other invertebrate taxa (Foret et al. 2012), and the correlation of predicted DNA methylation patterns and differential gene expression in *Acropora millepora* (Dixon et al. 2014) and other invertebrates (Roberts and Gavery 2012) provides ample rationale for assessing DNA methylation as a mechanism of phenotypic plasticity in reef‐building corals. Importantly, patterns of DNA methylation in the soma have the potential to be inherited through the soma, as sequestration of the germ cells is thought to be lacking in cnidarians (Buss 1983; but see Barfield et al. 2016) and some corals can produce asexual offspring (Yeoh and Dai 2010; Combosch and Vollmer 2013), thereby providing a memory of recent environmental change.

Generation of a temporal buffer through phenotypic plasticity is particularly important for organisms living near the edges of their performance envelope and those that are threatened by rapid rates of climate change, such as reef‐building corals (Reusch 2014). The anthropogenically induced increase in greenhouse gases in the atmosphere is predicted to increase sea surface temperature by ~2–3°C relative to pre‐industrial conditions and pCO_2_ up to ~930 ppm by 2100 (Pachauri et al. 2014), resulting in thermal extremes and ocean acidification (OA, a decline in pH driven by oceanic uptake of CO_2_ and shifting carbonate chemistry). Increased OA has been demonstrated to cause severe negative responses in corals. For example, OA increases the energetic cost of homeostasis of marine organisms (Pan et al. 2015), linked to impairment of calcification across a variety of species (Langdon and Atkinson 2005; Comeau et al. 2013). The ability to acclimatize to changing conditions is not only important for the reef‐building corals, but also to the diversity of life on reefs, and the goods and services they provide, which are valued on the order of billions of dollars annually (Bishop et al. 2012).

Early work in corals suggests that intragenerational beneficial acclimatization to environmental stressors occurs. For example, repeated bleaching of *Goniastrea* in Thailand resulted in acclimatization in the initially bleached portions of the corals, which did not display paling during the second event (Brown et al. 2002). Further, thermal history played a mitigating role in photophysiological performance of the coral symbionts preconditioned to increased temperature when they were subsequently re‐exposed (Middlebrook et al. 2008). More recently, work examining the mechanisms underlying beneficial acclimation to fluctuating temperatures identified strong ubiquitous expression of a set of genes that provided thermal resilience *via* expression frontloading of genes involved in heat‐shock response, antioxidants, and other regulatory roles (Barshis et al. 2013). Further, corals transplanted from a location of moderate thermal variability to that of high variability acquired the sensitivity of the transplantation destination, with gene expression profiles identifying a group of differentially expressed genes responsible for this acclimatization (Palumbi et al. 2014). These included genes involved, for example, in cell signaling, heat‐shock response, and those acting as molecular chaperones. There is also some indication that corals positively acclimatize to ocean acidification, with coral communities existing in the presence of low pH at CO_2_ seeps (Fabricius et al. 2011; Crook et al. 2013). Additionally, parental preconditioning to OA and temperature results in positive transgenerational acclimation in the offspring (Putnam and Gates 2015). Together, these studies indicate that phenotypic plasticity, which may provide rapid beneficial acclimatization to climate change, is a key mechanism for corals that has yet to be fully considered in our predictions reef futures in a time of rapid environmental change.

Despite the mounting evidence of the capacity for beneficial coral acclimation and acclimatization, studies of the mechanistic underpinnings are still in their infancy. Here, we tested DNA methylation as an epigenetic control mechanism underlying phenotypic plasticity in reef corals following exposure of clonal fragments to ambient and ocean acidification conditions. To test the hypotheses that environmentally induced DNA methylation is linked to plasticity in physiology, we exposed clonal fragments of *Montipora capitata* (Dana 1846) (resistant) and *Pocillopora damicornis* (Linnaeus, 1758) (sensitive) to ambient and high pCO_2_ conditions and measured changes in integrated cellular phenotype (metabolomic profiles), organism growth (calcification rates), and bulk coral DNA methylation. We choose these corals as a contrast between two coral species with demonstrated sensitivity to temperature and ocean acidification in the laboratory (Gibbin et al. 2015) and to temperature during natural bleaching events in the field (Bahr et al. 2015). Our results identify a stronger response of the sensitive coral species (*P. damicornis*) to OA and link phenotypic plasticity in response to ocean acidification to changes in DNA methylation, supporting a role of epigenetic control in the plasticity of corals. Our work infers that the induction of epigenetics and phenotypic plasticity may be a useful strategy for conservation and management through assisted evolution approaches (van Oppen et al. 2015) and highlights the need to further investigate DNA methylation as a mechanism of beneficial acclimatization.

## Materials and methods

### Coral collection and acclimation

Corals were collected from the fringing reefs of southern Kaneohe Bay (permit SAP2014 Hawaii DAR) in March 2014. Single genotypes of both *Montipora capitata* and *Pocillopora damicornis* found immediately adjacent to each other were used to ensure uniformity in host and symbiont genetics as well as environmental history. The temperature of the surrounding seawater was measured next to the corals (28 March–2 April, Fig. S1) before their transport to the Hawaii Institute of Marine Biology. The corals acclimated in tanks for 8 days were fragmented into nubbins (*n* = 30 per treatment for each species, see figure legends for response variable sample sizes), attached to plastic bases, and allowed to recover and acclimate in natural physical conditions within the acclimation tank (Fig. S1) for 24 days prior to allocation to the experimental treatments.

### Experimental design and setup

**Figure 1:**
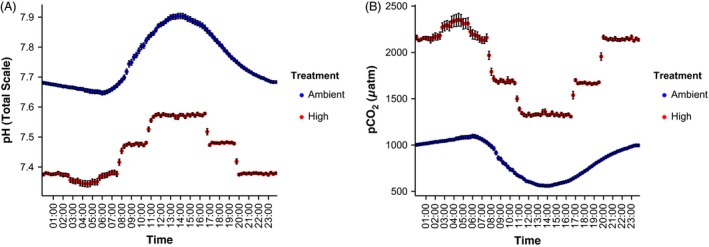
Average (mean ± SEM) diurnal cycle of experimental treatments in the common garden exposure tanks. (A) pH (NBS scale) was measured every 15 min in the tanks and converted to total scale (see Materials and methods for details). (B) pCO_2_ was calculated from pH (total scale) and average total alkalinity and salinity in each tank (see Materials and methods and Table 1 for details).

Ocean acidification treatments were produced in duplicate ~1300‐L common garden tanks where corals were held for the ~6 weeks (40 days) of experimental condition exposure following acclimation. A common garden approach was chosen to ensure identical conditions were maintained, along with holding genotype constant, in order to clearly test epigenetic response. These common garden tanks had a high water turnover rate (5× per day), thereby minimizing colony interactions. The tanks were shaded to ~60% full irradiance, and integrated light values were logged every 15 min with underwater logger (Odyssey PAR loggers standardized to Li‐Cor 192SA cosine sensor; Long et al. 2012; Figs S1 and S2). Temperature was logged every 15 min using underwater loggers (Hobo Water Temp Pro v2, accuracy = 0.21°C, resolution = 0.02°C, Onset Computer Corporation, Fig. S2). Coral fragments were held in common garden tanks at ambient (~560 to 1100 μatm) and high (~1320 to 2360 μatm) pCO_2_ (Fig. 1). The ocean acidification treatments were created using a pH‐stat system with a microprocessor‐controlled power strip (Apex Aquacontroller, Neptune Systems, Morgan Hill, CA, USA). The pH probe was calibrated weekly (NBS scale), and continuous measurement of pH was logged every 15 min. The pH feedback was used to guide the response of microprocessor‐controlled stainless steel solenoid valves (part 507731T1, McMaster Carr, Los Angeles, CA, USA) that injected ambient air or 99.9% food grade CO_2_ on demand through a venturi injector (MK‐484; Mazzei Injector Company LLC, Bakersfield, CA, USA) connected to a recirculating pump (700 gph Magnetic Drive; Danner Manufacturing Inc, Islandia, NY, USA). pH in the high pCO_2_ tank was offset from the natural fluctuation by 0.3 units by programming in different set points throughout the day with low variation about the desired set points (Fig. 1) for an approximate doubling of current pCO_2_ conditions. Additionally, the tanks were monitored ~daily by measuring temperature with a certified digital thermometer (5‐077‐8, accuracy = 0.05°C, resolution = 0.001°C; Control Company, Friendswood, TX, USA). pH was measured on the total scale with a handheld probe (DG115‐SC; Mettler‐Toledo, LLC, Columbus, OH, USA) calibrated against a Tris standard (A. Dickson certified reference material), and salinity (YSI 63; Yellow Springs Instruments, Yellow Springs, OH, USA) to further document the efficacy of the treatments generated by the pH‐stat system (Table 1).

**Table 1:** In addition to real‐time pH, light, and temperature measurements in each tank (15‐min frequency) and ~daily checks of temperature, salinity, and pH with handheld probes, seawater chemistry was assessed twice weekly for the 6 weeks that corals were in the treatment tanks (*n* = 12 per treatment, mean ± SEM). Carbonate parameters of the seawater (pCO_2_, CO_2_, ${\text{HCO}}_{3}^{-}$, ${\text{CO}}_{3}^{-2}$, DIC, aragonite saturation state) were calculated from measurement of temperature, pH (total scale), total alkalinity, and salinity using the seacarb package in r (see Materials and methods for more details)

Treatment	Salinity	Temperature, °C	pH, Total Scale	CO_2_, μmol kg^−1^	pCO_2_, μatm	${\text{HCO}}_{3}^{-}$, μmol kg^−1^	${\text{CO}}_{3}^{-2}$, μmol kg^−1^	DIC, μmol kg^−1^	Total Alkalinity, μmol kg^−1^	Aragonite saturation state
Ambient	34.0 ± 0.2	26.5 ± 0.2	7.99 ± 0.02	12 ± 1	453 ± 29	1700 ± 27	186 ± 9	1898 ± 20	2163 ± 10	3.0 ± 0.1
High	34.1 ± 0.2	26.5 ± 0.2	7.70 ± 0.02	27 ± 2	982 ± 58	1904 ± 12	107 ± 5	2038 ± 9	2171 ± 6	1.7 ± 0.1

### Carbonate chemistry

Carbonate chemistry was assessed according to the Guide to Best Practices (Riebesell et al. 2010) using standard certified reference materials (A. Dickson Laboratory, UCSD). Total alkalinity was measured twice weekly for each treatment as described in Putnam and Gates (2015) and did not differ significantly between ambient (2163 ± 17 μmol kg sw^−1^) and high (2171 ± 10 μmol kg sw^−1^) tanks. Continuous measurements of pH were converted from NBS to total scale using the equations from CO_2_Sys (Pierrot et al. 2006) implemented in r (v3.1.0, R Core Team 2014; https://github.com/hputnam/Coral_DNAMethylation_Plasticity). Carbonate parameters were calculated using the seacarb package (v3.0.11, Gattuso et al. 2015), with inputs from pH (total scale) at a 15‐min interval, using the average TA and salinity measured in each treatment tank (Table 1).

### Calcification

Calcification was assessed using the buoyant weight technique (Spencer Davies 1989) just prior to initiation of treatment conditions and every 2 weeks for the 6‐week duration of the experiment (i.e., weeks 2, 4, and 6). Aragonite density values for calculating dry weight were gathered from the literature for each genus and set as 2.78 g cm^−3^ for *P. damicornis* (Spencer Davies 1989; Al‐Sofyani and Floos 2013) and 2.03 g cm^−3^ for *M. capitata* (Anthony and Hoegh‐Guldberg 2003) for calculation of dry weight and of growth according to Spencer Davies (1989). Corals were weighed (Ohaus Adventurer Pro, AZ313, max = 310 g, 0.001 g) at each time point and normalized to the initial weight to obtain % calcification day^−1^. This method includes both calcification and dissolution process and thus represents a net response. The terms calcification and growth are used for comparability with the literature using this same technique (Spencer Davies 1989). Following examination of normality, square‐root‐transformed calcification data were analyzed with a repeated‐measures anova for the fixed factors of treatment (two levels) and species (two levels) using the lme function (nlme; Pinheiro et al. 2016) with random slopes and intercepts in the r statistical environment (v3.1.0, R Core Team 2014). *Post hoc* pairwise comparisons were completed with the contrast function (lsmeans; Lenth 2016) between treatments for each species, at each time point.

### ^1^H NMR metabolite profiling

A small fragment of coral tissue and skeleton was removed from each sample and lyophilized for 24 h. From each lyophilized sample, ~0.1 g was weighed on an analytical balance (Mettler‐Toledo XS205 Dual Range, max 81 g, d = 0.01 mg) and placed in 2 mL of 70% HPLC grade methanol and 30% HPLC grade water and sonicated for 15 min in an ice water bath and shaken on an orbital shaker (Fisher Scientific, Pittsburgh, PA, USA) at ~130 rpm at 4°C for 24 h. After 24 h, the solvent was removed and stored at −80°C. Metabolite extraction was repeated three times based on a preliminary experiment that identified exhaustive extraction after 3 days for both species. The full extract volume was spun at 4000 rpm at 4 °C to pellet any debris. An aliquot of 1 mL was dried in a speed vacuum concentrator at room temp for ~6 h and the extract weighed on the analytical balance and stored at −80°C overnight. Samples were resuspended in 250 μL of heavy water (deuterium oxide; D_2_O) with a standard spike of 1 mm sodium 3‐(trimethylsilyl)propionate 2,2,3,3‐d4 (TMSP), sonicated for 15 min in an ice water bath, and added to 3 mm ^1^H NMR tubes for measurement.

Resuspended extracts were run on a 500 MHz Varian Unity Inova spectrometer with a 1 m/x‐broadband 3‐mm probe according to Sogin et al. (2014), with 132 transients. All spectra were imported into mestrenova (v7.1.2 Mestrelabs, Escondido, CA, USA) and quality controlled manually using phase correction, baseline correction (full auto Whittaker smoother), and zeroing of the TMSP peak. Spectra from all samples were aligned prior to binning, and each spectrum was processed with integral normalization to area under the curve resulting in a relative quantity for each bin, defined here as an individual metabolite data point. Bins were assigned at 0.04‐ppm intervals between 0.5 and 10 ppm and exported as ASCII files. Bins containing the TMSP peak were excluded by the truncation of data at 0.5 ppm, and bins containing the water peak (4.73959–4.93955 ppm) were excluded manually prior to multivariate analysis.

Analyses to test for significant differences between species and treatments were carried out in the r statistical environment (v3.1.0, R Core Team 2014). First, bins were normalized to extract weight to enable direct comparison across samples and negative values corrected to zero. The percent relative standard deviation (%RSD) was calculated for each species and treatment group to compare variability between groups. For multivariate analysis, data were centered and scaled using pareto scaling to increase the weight of intermediate peaks relative to high peaks while minimizing baseline noise in the spectra. Principal components analysis was used to assess outliers, which were removed outside the 99% confidence interval limits. Data were analyzed using orthogonal partial least squares–discriminate analysis (OPLS‐DA, Sogin et al. 2014; https://github.com/Anderson-Lab/OPLS) at the level of species (independent of treatment) and at the level of treatment within each species following a significant separation of species. The OPLS‐DA approach was used to quantify loading values describing the contribution of each metabolite bin to the model. A statistical total correlation spectroscopy (STOSCY) method was used to calculate correlations between the bins that are significant drivers of the OPLS‐DA separation in species or treatments and all other metabolite bins. The resulting values provide the locations of high correlation that can be used to assist in multipeak metabolite identification. The correlation analysis results and visualization of the spectra were used in combination to assist identification of metabolites against Chenomx 500 MHz spectral libraries (Sogin et al. 2014). Representative spectra for all species and treatments were assessed for the identity of all peaks >0.02 intensity, or >1% of the TMSP peak (1 mm). Full statistical analysis is available at https://github.com/hputnam/Coral_DNAMethylation_Plasticity.

### DNA Methylation

Host and symbiont fractions were separated using centrifugation based on modifications from Papina et al. (2003) to further minimize fraction carryover. Briefly, for each sample separately, tissue was airbrushed into a slurry and homogenized using a sterilized glass homogenizer. The homogenate (10 mL) was subject to centrifugation (600 *g* for 5 min at 4°C) to pellet algal cells. Care was taken to remove only the supernatant (initial – 1 mL) so as not to disrupt the algal pellet. The centrifugation and removal was repeated 5 times for a final volume of 5 mL. Finally, 300 μL was removed from the top portion of the last centrifugation and used for host DNA extraction.

Genomic host DNA was extracted from each sample following separation using a CTAB extraction protocol (protocols.io dx.doi.org/10.17504/protocols.io.dyq7vv; Baker and Cunning 2016), and DNA was quantified spectrophotometrically. Whole‐genome DNA methylation of the coral host fraction was assessed colorimetrically in duplicate using a methylated DNA immunoprecipitation assay according to manufacturer's instructions (MethylFlash Methylated DNA Quantification kit, P‐1034 Epigentek, Farmingdale, NY, USA) and reported as % methylated DNA, relative to the input DNA quantity for each coral sample (see Fig. 3 legend for sample size). DNA methylation data were analyzed with two‐way anova following transformation (4th root) to meet the assumptions of normality and homogeneity of variances, in the r statistical environment (v3.1.0, R Core Team 2014).

## Results

Temperature was measured at the site of collection (28 March–2 April) and ranged from 24.6 to 27.0°C. This natural cycle of thermal fluctuation was maintained in the experimental tanks during acclimation and treatment (Figs S1 and S2), with an average of 26.54 ± 0.01°C (mean ± SEM) logged during the experimental exposure. The natural cycle of pH and pCO_2_ fluctuation was also maintained in the tanks, with low variability around the programmed conditions (Fig. 1). Natural lighting was used and fluctuated throughout the day, with a diel range of ~235 μmol m^−2^ s^−1^.

### Metabolomic profiling

**Figure 2:**
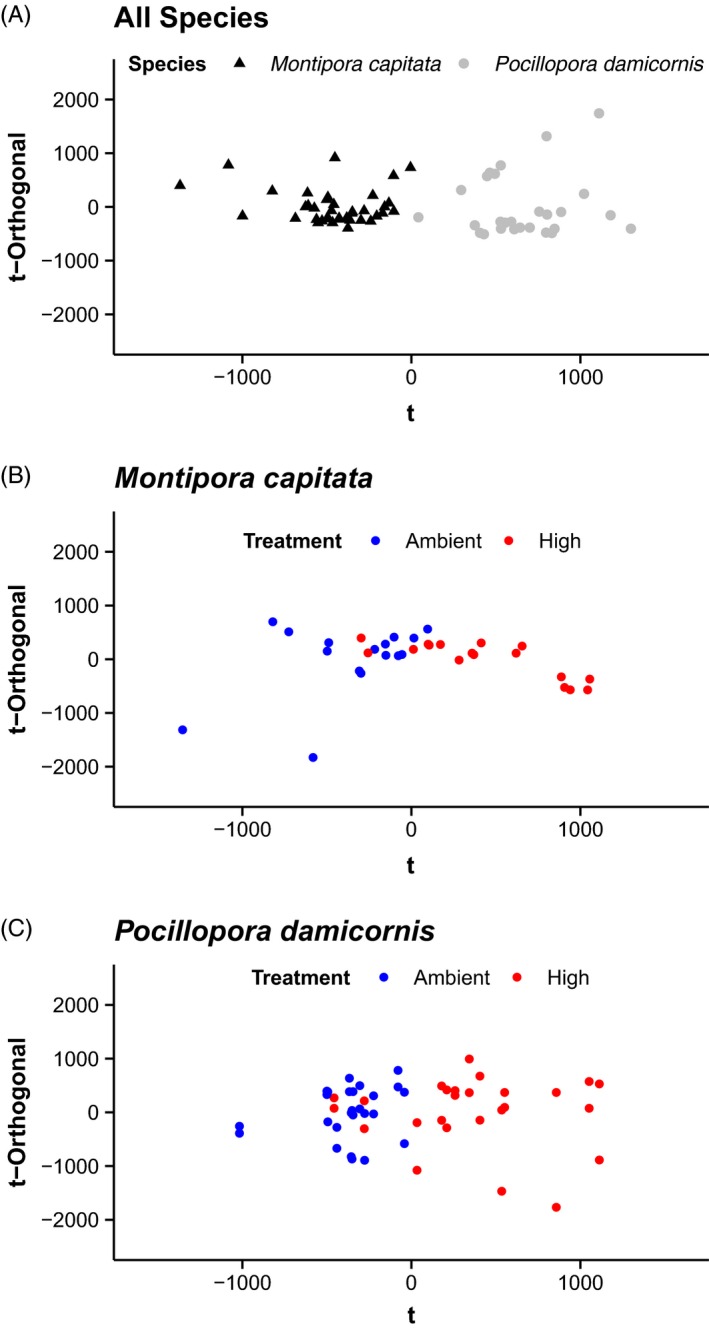
OPLS‐DA models of ^1^ H‐NMR metabolomic profiles for (A) coral species (*n* = 39 for *Montipora capitata*,* n* = 27 for *Pocillopora damicornis*), (B) by treatment for *M. capitata* samples (*n* = 17 for ambient, *n* = 17 for high), and (C) by treatment for *P. damicornis* samples (*n* = 13 for ambient, *n* = 13 for high). See text for model statistical results.

**Figure 3:**
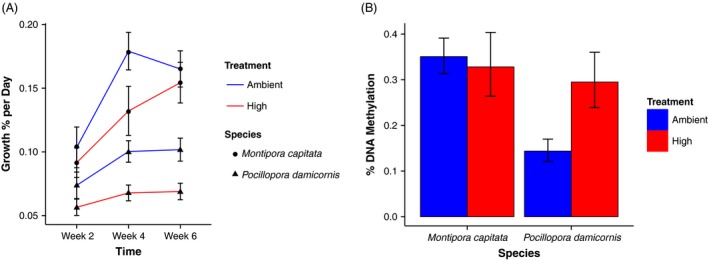
(A) Coral growth (% per day relative to initial mass) measured using the buoyant weight technique (Spencer Davies 1989) for replicate clonal fragments of both coral species in ambient and high pCO_2_ conditions (*n* = 22 for each *Pocillopora damicornis* point and *n* = 25 for each *Montipora capitata* point except at week 6, where *n* = 24 for *M. capitata* high pCO_2_). This method includes both calcification and dissolution process and thus represents a net result. Data shown are back‐transformed and statistical contrasts in Table 2. (B) DNA methylation (% of total DNA) of the coral host measured colorimetrically for replicate clonal fragments of both coral species in ambient and high pCO_2_ conditions (*n* = 8, 8, 7, 8 from left to right). There was a significant Species by Treatment interaction in % DNA methylation following exposure for 6 weeks (*P *= 0.05; Table 2).

Metabolomic profiles were considered in this study to provide an integrated response across multiple biological pathways into an assessment of cellular phenotype. Equal variation (%RSD) in metabolomic profiles was present in all species and treatment groups (Kruskal–Wallace *χ* ^2^ = 5.3923, df = 3, *P *= 0.145) indicating equal variability in the clonal fragments (Fig. S3). Multivariate discriminate analysis (OPLS‐DA) identified strong capacity for discrimination of the metabolite profiles between species (*Q* ^2^ = 0.745, *P *< 0.01, Fig. 2). The identified metabolites were primarily represented by carboxylic acids (acetate and formate), fatty acids (azelate, caprate, caprylate, sebacate, suberate, glycerol), amino acids (glutamate, aspartate, betaine, glycine, proline), and monosaccharides (glucose; Table S1). There were 129 bins that contributed significantly to the separation in species profiles (Table S1). Only 11.6% of the bins could be identified as unique against the metabolite database (Chenomx 500 MHz spectral libraries). Based on the significant species level differences in profiles, species were separated for subsequent treatment comparisons. When testing the effect of ocean acidification exposure relative to controls within each species, *P. damicornis* displayed a much stronger discrimination capacity (~2×) in the metabolite profile between treatments (*Q* ^2^ = 0.291, *P *< 0.01, Fig. 3) than *M. capitata* (*Q* ^2^ = 0.137, *P *< 0.04, Fig. 3). There were 31 metabolite bins that contributed most strongly to the separation in metabolomics profiles for *P. damicornis* (Table S2). *M. capitata* spectra showed greater complexity in treatment response, with 71 metabolite bins contributing most strongly to the separation in metabolomics profiles (Table S3). In general, exposure to high pCO_2_ resulted in a decline in metabolite quantity, where 98.6% and 96.8% of bins in *M. capitata* and *P. damicornis*, respectively, had higher values in the ambient treatment compared to the high. Extensive attempts were made for the identification of individual metabolites, but given the lack of coral metabolite databases, there was a low success rate of individual unique metabolite identification against the existing reference database (8.5% for *M. capitata* and 25.8% *P. damicornis*), as reported previously for ^1^H NMR work (Sogin et al. 2014). The lack of ability to annotate metabolite bins precluded any further pathway or network analysis at this time. Assessment of the identity of all peaks with >0.02 intensity or >1% of the TMSP peak (1 mm) is reported for representative spectra from both species and treatments (Table S4).

### Calcification

Coral calcification rates varied over the course of the experiment (Table 2, *P* < 0.0001). On average, the calcification of *M. capitata* was 1.8× higher than *P. damicornis* (*F*_1,90_ = 32.59, *P *< 0.0001, Fig. 3A). Both species responded negatively to the high pCO_2_ treatment (*F*_1,89_ = 4.81, *P *= 0.031), resulting to no interactive effects between treatment and species (Table 2, *P *> 0.05). Pairwise contrasts of treatments at each time point identified stronger significant decline in *P. damicornis* (Table 2B, *P *≤ 0.05) at high pCO_2_ at weeks 4 and 6, compared to ambient, whereas *M. capitata* high pCO_2_ only differed significantly from ambient at week 4 (Table 2B, *P *< 0.05).

**Table 2:** (A) Repeated‐measures anova results of coral calcification, with (B) statistical contrasts, and (C) and two‐way anova results of coral DNA methylation

(A) Growth (sqrt (value − 1))						
Source	num df	den df		*F*		*P*
Intercept	1	179		211231		**<0.0001**
Time	2	179		27.23		**<0.0001**
Treatment	1	90		4.81		**0.0309**
Species	1	90		32.59		**<0.0001**
Time × Treatment	2	179		2.67		0.0718
Time × Species	2	179		7.26		**0.0009**
Treatment × Species	1	90		0.34		0.5607
Time × Trt × Sp	2	179		1.57		0.2106

(B) Statistical contrasts of growth between ambient and high treatments for each species at each time point. See Materials and methods for details						
Timepoint	*Montipora capitata*			*Pocillopora damicornis*		
	*t*	df	*P*	*t*	df	*P*
Week 2	0.72	90	0.47	1.12	90	0.26
Week 4	2.52	90	0.01	1.92	90	0.06
Week 6	0.65	90	0.52	2.15	90	**0.03**

(C) Methylation (fourth root)						
Source	df	SS	MS	*F*		*P*
Treatment	1	0.01869	0.01869	2.2431		0.14582
Species	1	0.05524	0.05524	6.6312		**0.01582**
Trt × Sp	1	0.03461	0.03461	4.1539		**0.05144**
Residuals	27	0.22494	0.00833			

Bold text indicates statistical significance with *P* < 0.05.

### DNA Methylation

Species displayed strong differences in their DNA methylation in response to treatment (*F*_1,27_ = 4.15, *P *= 0.05). Specifically, there was no difference in host DNA methylation in *M. capitata* between treatments at week 6 and an approximate doubling of methylation in the high pCO_2_ treatment in comparison with the ambient for *P. damicornis* (Table 2, Fig. 3B). The % DNA methylation within the host fraction of *M. capitata* was ~2.4 times higher than in *P. damicornis* (*F*_1,27_ = 6.63, *P *= 0.016, Fig. 3) within the baseline ambient condition.

## Discussion

Mechanisms of rapid adaptation and acclimatization of corals are of primary concern for the maintenance of diverse and functional reef ecosystems in a future of a rapidly changing climate (van Oppen et al. 2015). The induction and heritability of epigenetic modifications and their evolutionary consequences are key to mitigating the discrepancy between phenotype and environment (Rodríguez‐Romero et al. 2015; Chakravarti et al. 2016). Our results indicate inducible DNA methylation provides one such avenue for generating phenotypic plasticity, but that response is likely to vary by taxa and duration of exposure. This variation speaks to potential mechanisms for differential performance under environmental stressors among coral species (Loya et al. 2001). Furthermore, our findings provide a basis for future testing of the heritability and longevity of epigenetics for use in assisted evolution endeavors (van Oppen et al. 2015), as an acclimatory buffer against climate change in a conservation context.

Phenotypic plasticity manifests at various levels of biological organization. One primary level with rapid response is the metabolic or biochemical level. Here, metabolomic profiling provides a molecular phenotype of the organism by quantifying the intermediates and products of many biochemical processes. Metabolomic profiling has only recently been applied to corals (Gordon et al. 2013; Sogin et al. 2014) and their dinoflagellate symbionts (Klueter et al. 2015). This initial application was primarily concerned with methodology and reproducibility, but did identify differences in the metabolite profiles between coral species (Sogin et al. 2014), between *Symbiodinium* types in culture (Klueter et al. 2015), and between samples exposed to mechanical stress versus controls (Gordon et al. 2013). These metabolites include amino acids, lipids, sugars, and other small molecules, which are important in the performance of each of the holobiont partners separately and are also key players in the nutritional recycling in the coral–dinoflagellate symbiosis (Gordon and Leggat 2010). Prior ^1^H NMR analyses have suffered from low metabolite identification capacity (~10%, Sogin et al. 2014). Here, we were also only able to identify a maximum of 26% of unique metabolites in any comparison. Among those identified, we found metabolites in general functional groups of carboxylic acids, fatty acids, amino acids, and monosaccharides, which supports prior metabolite identification from corals (Gordon and Leggat 2010; Sogin et al. 2014). For all of the metabolites identified in this study (and the majority of metabolite bins contributing to model separation, >95%), there were significantly lower amounts of metabolites present under exposure to high pCO_2_. This reduction under OA conditions may indicate a general suppression of metabolic activities, as has been demonstrated for marine invertebrates exposed to reduced pH (Pörtner 2008). While metabolic depression can act as a protective mechanism with energetic benefits, it comes at cost of protein maintenance and production (Hand and Hardewig 1996), which could have negative implications for epigenetic regulation by impeding *de novo* and maintenance methyltransferase expression and activity.

While the lack of coral specific databases hampered our individual metabolite identification, we were still able to demonstrate the effectiveness of metabolic phenotyping of the coral holobiont (host and eukaryotic and prokaryotic symbionts) following exposure to environmental perturbation (i.e., OA). The application of metabolomic profiling to clonal fragments clearly demonstrates plasticity in the biology in response to the OA treatment, which is stronger in *Pocillopora damicornis* than *Montipora capitata* at the time point sampled. It is now critical to continue with detailed approaches to identify the location of changes in methylation in the genome and the downstream pathways. This will require substantial investment in improving metabolomic databases, as well as the assessment of other levels of biological response, such as gene expression, where a direct link has been made between expression plasticity and regulation by DNA methylation (Feil and Fraga 2012).

At a higher level of biological organization, calcification of the clonal fragments also displayed plasticity in response to the OA treatments. Similar to the metabolomic profiling, *P. damicornis* displayed a stronger calcification change when exposed to high pCO_2_. The finding of relatively weak change in calcification in *M. capitata* and stronger decline in *P. damicornis* under high pCO_2_ is consistent with results from prior studies in Hawaii. A mesocosm exposure of *M. capitata* to ~700 to 1600 μatm pCO_2_ resulted in declines of ~15% in calcification relative to ambient (Jokiel et al. 2008), whereas exposure of *P. damicornis* to 1000 μatm pCO_2_ resulted in ~28% decline in calcification compared to ambient pCO_2_ (500 μatm, Comeau et al. 2014). The acclimation of *M. capitata* calcification and DNA methylation to control levels by week 6, while *P. damicornis* maintained differences in both factors, suggests that there could be a direct role for DNA methylation of biomineralization control. The comparison of DNA methylation associated with calcification genes in corals with differing acclimatization dynamics provides a fruitful area of investigation given the concern for biomineralization processes under increasing ocean acidification (Doney et al. 2009).

Homeostatic controls to modulate response to a heterogeneous environment are universal within the metazoans. These controls are regulated at the local level in response to direct environmental stimuli, but organisms differ their umwelt (Von Uexkull 1909), or the way they experience the environment, or detect and transduce external signals (Van Dyck 2012). Comparison of the biology of the sensitive *P. damicornis* in comparison with *M. capitata* reveals a variety of differences associated with, and perhaps contributing to, their differential environmental experience. *Pocillopora* is an imperforate coral with a thin layer of tissue directly over the skeleton, providing less habitat for symbionts and less tissue biomass for energy reserves (Yost et al. 2013). In contrast, *Montipora* is a perforate coral with thick tissues that extend down into the skeleton in a complex matrix (Yost et al. 2013). This tissue thickness provides a sizeable habitat for their symbionts, as well as a storehouse for energetic compounds in the form of proteins, lipids, and carbohydrates (Rodrigues and Grottoli 2007) that are 2.7, 3, and 16.3 times higher than those in *P. damicornis*, respectively (Achituv et al. 1994). *Montipora* and other thick‐tissued corals may have some capacity to buffer the intracellular environment from external environmental change (Jimenez et al. 2008; Gibbin et al. 2015). For example, work by Gibbin et al. (2015) that manipulated the external seawater pH found that *M. capitata* can maintain its intracellular pH following exposure to increased temperatures, but the intracellular pH of *P. damicornis* under increased OA declines significantly when the temperature is increased and corals are bleached.

These physiological differences suggest *Montipora* may receive more buffering from the external environment or perceive it in a more coarse‐grain fashion, whereas *Pocillopora* is less internally buffered and the external environmental perception may be more fine grained (Selander and Kaufman 1973). It is therefore possible that OA does not induce *de novo* DNA methylation in *M. capitata* due to the buffering capacity of the thick tissues (Jimenez et al. 2008; Yost et al. 2013). With this physiological tissue buffer in *Montipora*, the plasticity in control of gene expression is not required for a high magnitude of environmental responsiveness; hence, higher baseline DNA methylation that is not as responsive to external stimuli. As *P. damicornis* does not have the physiological characteristics that support resistance to the heterogeneous and dynamic environmental change, DNA methylation may be lower globally to provide the capacity for dynamic acclimatization, similarly to the response capacity suggested by the predicted methylation patterns of *Acropora* (Dixon et al. 2014) and oysters (Gavery and Roberts 2010). The increase of methylation in *P. damicornis* following exposure to OA may then provide necessary homeostatic control through changes in gene expression, such as a higher constitutive expression of a variety of genes (i.e., frontloading; Barshis et al. 2013).

Conversely, it is possible that the internal environment of *Montipora* is more extreme than *Pocillopora* and generates a stronger internal signal than the external oscillations, requiring high methylation that generates more ubiquitous expression of environmentally responsive genes to deal with internal physical dynamics, damping external responsiveness. For example, under the same irradiance, a thick‐tissued perforate skeleton coral (massive *Porites* sp.) warmed almost 1°C above seawater, whereas the thin‐tissued, imperforate Pocilloporid (*Stylophora pistillata*) warmed <0.5°C (Jimenez et al. 2008). Oxygen concentration and pH differ substantially as a function of tissue depth and interact with the irradiance regime (Kuhl et al. 1995). Additionally, differences in tissue–skeletal characteristics result in varied optical microhabitats that can drive strong physico‐chemical environments within the tissues (Enríquez et al. 2005; Wangpraseurt et al. 2012; Wangpraseurt et al. 2016). This type of extreme internal environment may then require high DNA methylation that necessitates more global expression of environmentally responsive genes to maintain homeostasis.

An additional hypothesis for the differences between the DNA methylation in *M. capitata* and *P. damicornis* is the potential for complete acclimation, or return to control levels in *Montipora* (Allan et al. 2014), which could occur through physiological adjustments at the molecular level through short‐term modifications of existing resources (Hochachka and Somero 2002). Based on the calcification dynamics (Fig. 3) and similarity between ambient and high treatments for *M. capitata* at week 6, complete acclimation appears to be a possible scenario. This potential for complete acclimation does not, however, discount our preceding hypotheses, as baseline levels of DNA methylation still differ by a factor of ~2 between the two species, suggesting a role for morphology and physiology as environmental buffers. The contrast of partial and complete acclimation at the same time point highlights the need to assess methylation dynamics under a range of environmental stressors. Future time series work pairing bisulfite sequencing to detect DNA methylation with RNASeq to identify expression patterns associated with the methylation will provide information to correlate with internal and external environmental fluctuations, identify the mechanistic linkages, and clarify methylation dynamics for a variety of species.

The field of environmental epigenetics includes examples of epigenetic regulation of gene expression associated with differential DNA methylation driven by changes in physical environment (Dowen et al. 2012), parental care (Weaver et al. 2004), and diet (Cooney et al. 2002), among others. For example, *Arabidopsis thaliana* exposure to bacterial pathogens drives differential methylation in gene‐rich regions of the genome supporting the hypothesis of epigenetic regulation of gene expression through DNA methylation (Dowen et al. 2012). Additionally, differences in parental care through licking and grooming of rat offspring induced changes in DNA methylation associated with the promoter of the glucocorticoid receptor resulting in downstream influences on gene expression with behavioral consequences (Weaver et al. 2004). These studies among others suggest substantial potential for environmentally induced intragenerational and transgenerational acclimatization.

Initial work in marine taxa identifies a role for adaptive transgenerational acclimation in response to OA at the organismal level (Miller et al. 2012; Parker et al. 2012; Allan et al. 2014; Lane et al. 2015). For example, work by Miller et al. (2012) demonstrated acceleration of routine metabolic rate (RMR) when naïve coral reef fish were challenged with OA conditions. Conversely, when parents were preconditioned to high CO_2_, exposure of offspring demonstrated metabolic acclimation, or compensation of RMR, to the level of controls (Miller et al. 2012). Specifically, in terms of corals, preconditioning of *P. damicornis* adults to increased temperature and OA during the brooding period results in metabolic acclimation of the larvae when exposed to the simulated future conditions a second time (Putnam and Gates 2015). Additionally, in an ecological context, survivorship and growth are higher in *P. damicornis* juveniles from parents preconditioned to high pCO_2_ when re‐exposed to OA conditions (H. M. Putnam, unpublished data). The induction of differential DNA methylation that we demonstrate here supports a role for environmentally induced DNA methylation in coral transgenerational acclimation and the heritability of this mechanism is now being tested *via* bisulfite sequencing of across generations.

In regard to the role of epigenetics in ecological and evolutionary processes, a primary hypothesis for the role of adaptive plasticity through DNA methylation is the enhancement of persistence in novel environmental conditions. It is, however, not just the direction of the plasticity but changes in mean and variance of the plasticity that are important under new conditions (Ghalambor et al. 2007), providing the capacity to facilitate adaptive evolution. For example, changes in DNA methylation induced by the physical environment can generate plasticity as a substrate for selection on the epigenetic system (Ghalambor et al. 2007; Flores et al. 2013). Additionally, phenotypic plasticity generated by epigenetic changes has potential to become genetic variation through genetic accommodation (Pigliucci 2006; Wund 2012). Further, plasticity generated at the parental level may be maladaptive, but result in adaptive transgenerational acclimation (Putnam and Gates 2015). DNA methylation can therefore be viewed as a rapid and dynamic mechanism that facilitates fine‐tuning in response to novel physical environments. It may not always result in complete acclimatization relative to control values, but it has an important role nonetheless.

## Conclusions and applications

Our results support the finding that OA is an environmental signal that triggers phenotypic plasticity in corals. Species‐specific differences in DNA methylation may result from differential umwelt, sensitivity to OA and other environmental stressors *via* physical, morphological, symbiotic, or physiological buffers, or differences in temporal acclimation dynamics. Our work suggests a role for *de novo* DNA methylation as a driving mechanism for phenotypic plasticity that may underlie intra‐ and transgenerational acclimation, and the heritable nature of this DNA methylation has been clearly demonstrated in other taxa (Verhoeven et al. 2010; Mirouze and Paszkowski 2011; Schield et al. 2015). The environmental induction of DNA methylation and adaptive plasticity in corals may provide an opportunity for assisted evolution of corals facing rapid climate change (van Oppen et al. 2015), much as hardening has been used to increase terrestrial crop and marine fisheries resilience and yield (Farooq et al. 2006; Chandra et al. 2010; Stevens 2014). For example, preconditioning or hardening against physical environment could be used to increase environmental tolerance (Brown et al. 2002), or identification of heritable epialleles could facilitate the outcomes of selective breeding programs (Hauser et al. 2011). Further determination of the extent, heritability, and longevity of epigenetic mechanisms in corals is therefore warranted. Identifying the genomic location and resulting transcriptional control throughout whole genomes is a critical next step in assessing the importance of the role of epigenetics in adaptation of corals to rapid climate change through soft inheritance.

## Data archiving statement

All data and analysis scripts have been deposited in Dryad (datadryad.org) under the following DOI: http://dx.doi.org/10.5061/dryad.nn8kv.

The data are also available at https://github.com/hputnam/Coral_DNAMethylation_Plasticity.

## Supporting information


**Figure S1.** Average (mean ± SEM) diurnal cycle of field and tank acclimation period measured every 15 min for (A) field temperature (*n* = 679) and (B) tank acclimation temperature (*n* = 3254), and (C) tank irradiance (*n* = 1440).

**Figure S2.** Average diurnal cycle (mean ± SEM) of experimental treatments in the common garden exposure tanks measured every 15 min for (A) temperature (*n* = 3517) and (B) irradiance (*n* = 1989 for ambient and *n* = 1990 for high), with light only reported during daylight interval ~5:45–19:45).

**Figure S3.** Boxplot displays of percent relative standard deviation (%RSD) of metabolite profiles across all metabolite bins from each species and treatment combination.

**Table S1.** Metabolite bins driving separation in profiles between *M. capitata* and *P. damicornis*.

**Table S2.** Metabolite bins driving separation in profiles between High and Ambient treatments for *M. capitata*.

**Table S3.** Metabolite bins driving separation in profiles between High and Ambient treatments for *P. damicornis*.

**Table S4.** Metabolite identification of representative spectra for all species and treatments as assessed for the identity of all peaks >0.02 intensity, or >1% of the TMSP standard peak (1 mm).
