# Supplementary figures and images for: Ocean acidification influences host DNA methylation and phenotypic plasticity in environmentally susceptible corals
Source: Evol Appl. 2016 Aug 2;9(9):1165–78. doi: 10.1111/eva.12408 (PMC5039329; doi:10.1111/eva.12408)

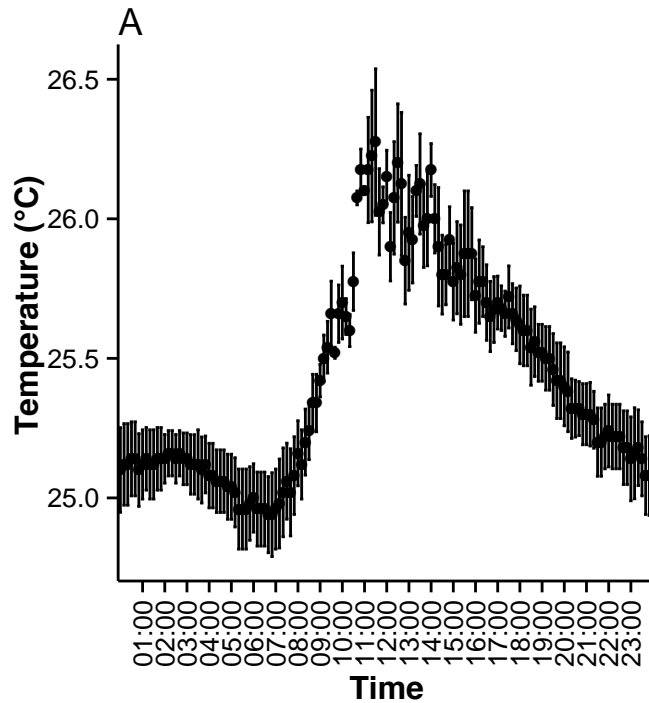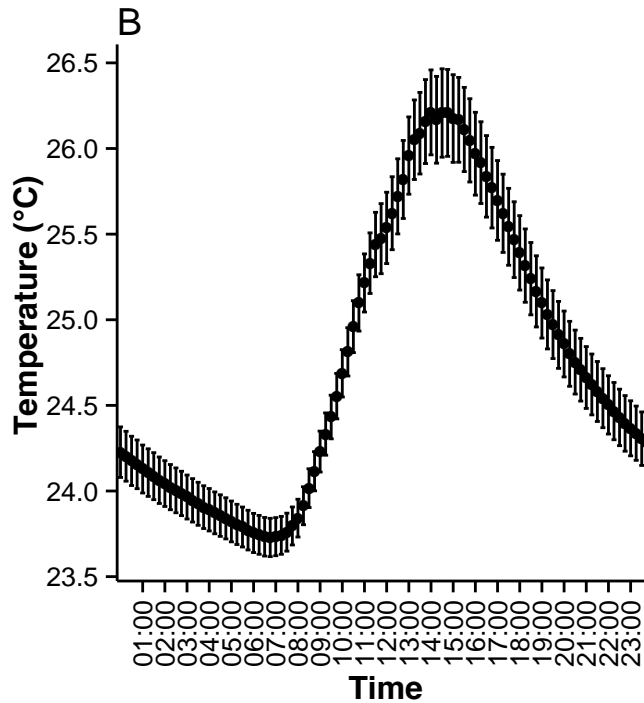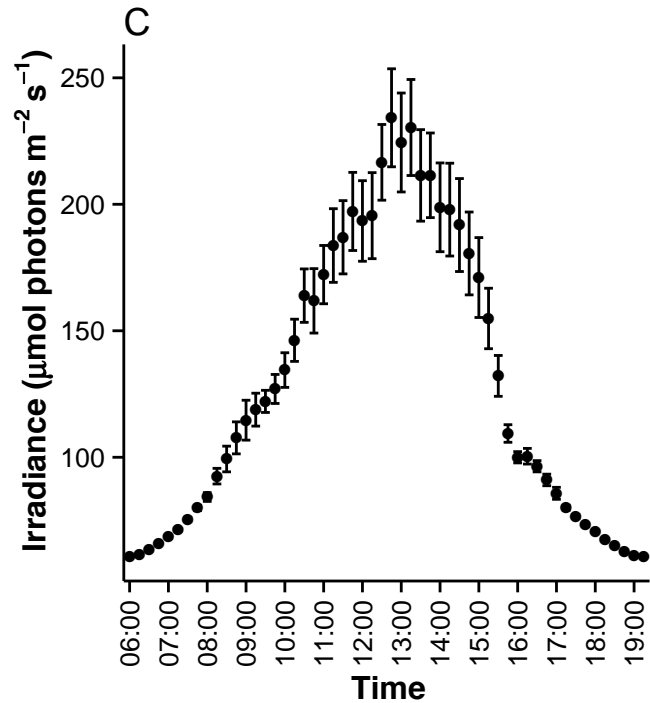

Supplement: Supplementary file 1 — Figure S1. Average (mean ± SEM) diurnal cycle of field and tank acclimation period measured every 15 min for (A) field temperature (n = 679) and (B) tank acclimation temperature (n = 3254), and (C) tank irradiance (n = 1440). [file EVA-9-1165-s001.pdf]

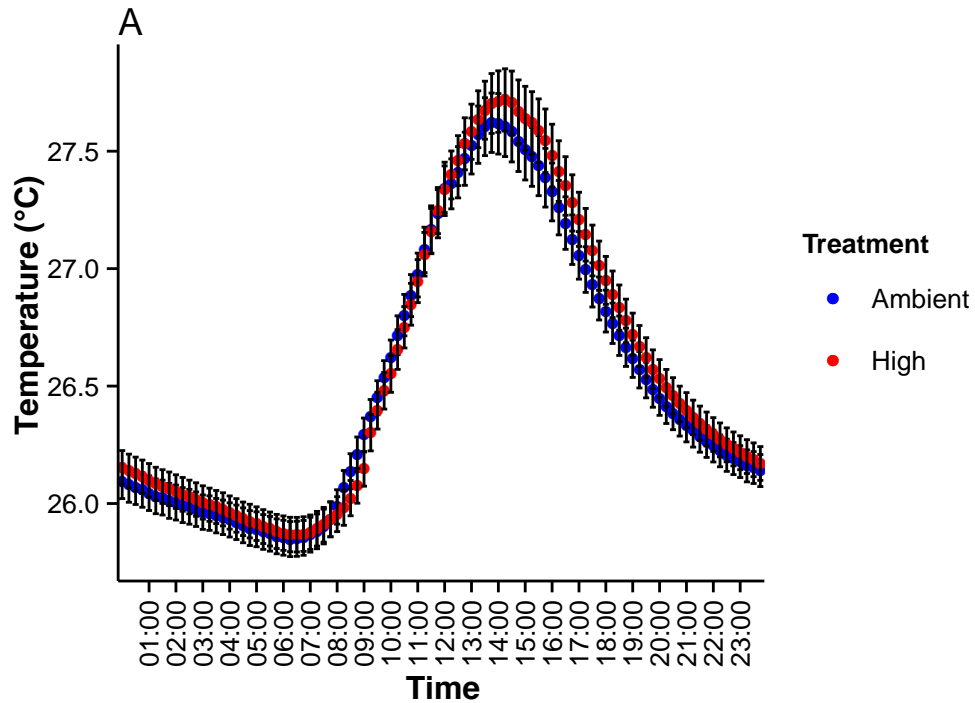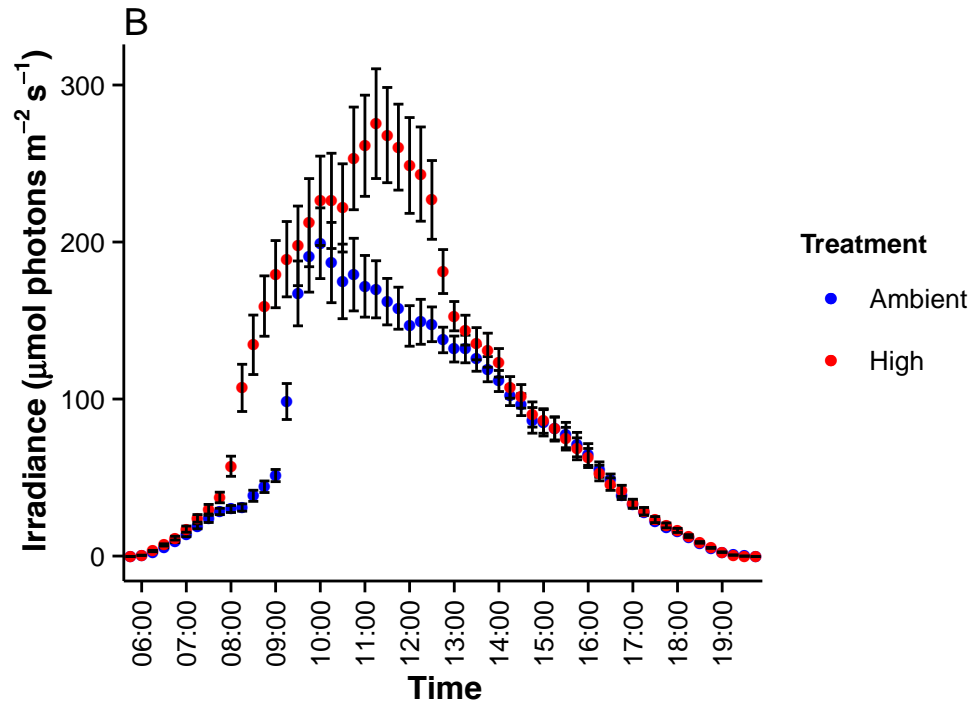

Supplement: Supplementary file 2 — Figure S2. Average diurnal cycle (mean ± SEM) of experimental treatments in the common garden exposure tanks measured every 15 min for (A) temperature (n = 3517) and (B) irradiance (n = 1989 for ambient and n = 1990 for high), with light only reported during daylight interval ~5:45–19:45). [file EVA-9-1165-s002.pdf]

% RSD

500  
400  
300  
200  
100  
0

*M. capitata*

*M. capitata*

*P. damicornis*

*P. damicornis*

Treatment

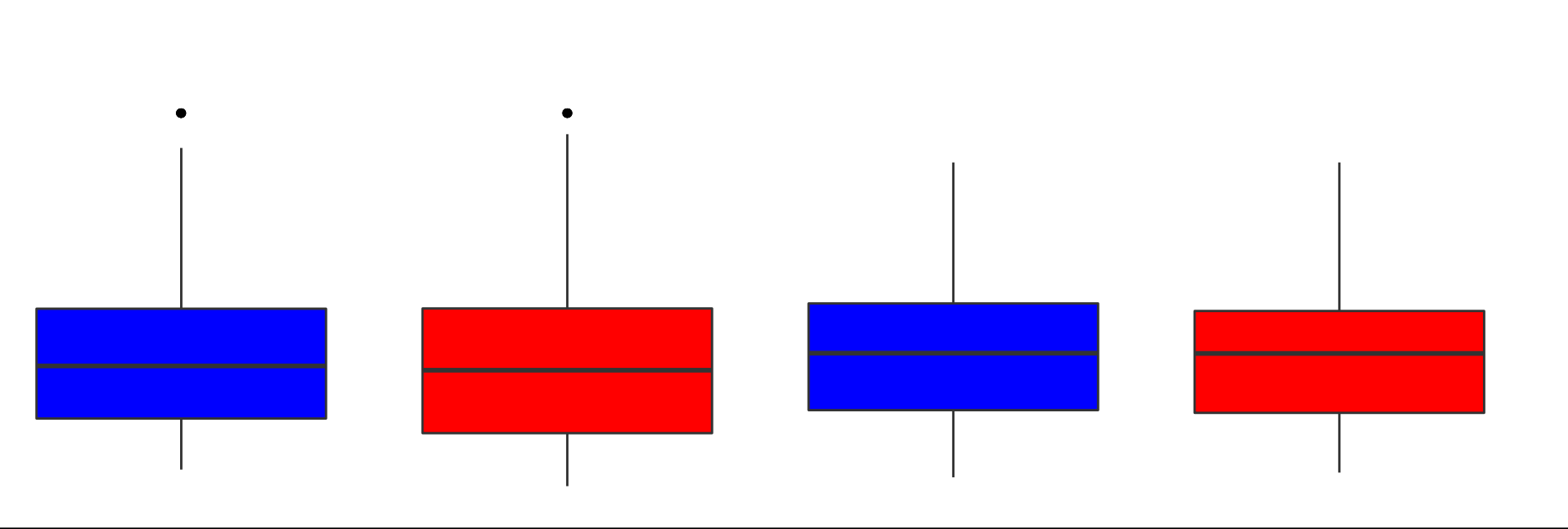

Supplement: Supplementary file 3 — Figure S3. Boxplot displays of percent relative standard deviation (%RSD) of metabolite profiles across all metabolite bins from each species and treatment combination. [file EVA-9-1165-s003.pdf]
